# Supplementary material for: Inter-laboratory automation of the in vitro micronucleus assay using imaging flow cytometry and deep learning
Source: Arch Toxicol. Author manuscript; Available in PMC 2022 Mar 1. (PMC8380241; doi:10.1007/s00204-021-03113-0)
Supplement: Supplementary Information [file NIHMS1732504-supplement-Supplementary_Information.pdf]

Supp. Table 1 – Imaging flow cytometry data acquisition information

Centre	Excitation laser (nm)	Intensity (mW)	Brightfield channel	Nuclear fluorescence channel	Nuclear stain	Objective lens	Cytometer Model
Cambridge	405	50	Ch04	Ch01	Hoechst 33342	40X	Amnis ImageStream^{X}
Cardiff	488	100	Ch01	Ch11	DRAQ5	40X	Amnis ImageStream^{X} MkII
GSK	642	55	Ch01	Ch11	DRAQ5	40X	Amnis ImageStream^{X} MkII

Image data were collected using three different imaging flow cytometers located across three laboratories (Cambridge, Cardiff and GSK). At each laboratory, the choice of florescent nuclear stain depended upon local protocols and compatibility with the cytometer's laser configuration.

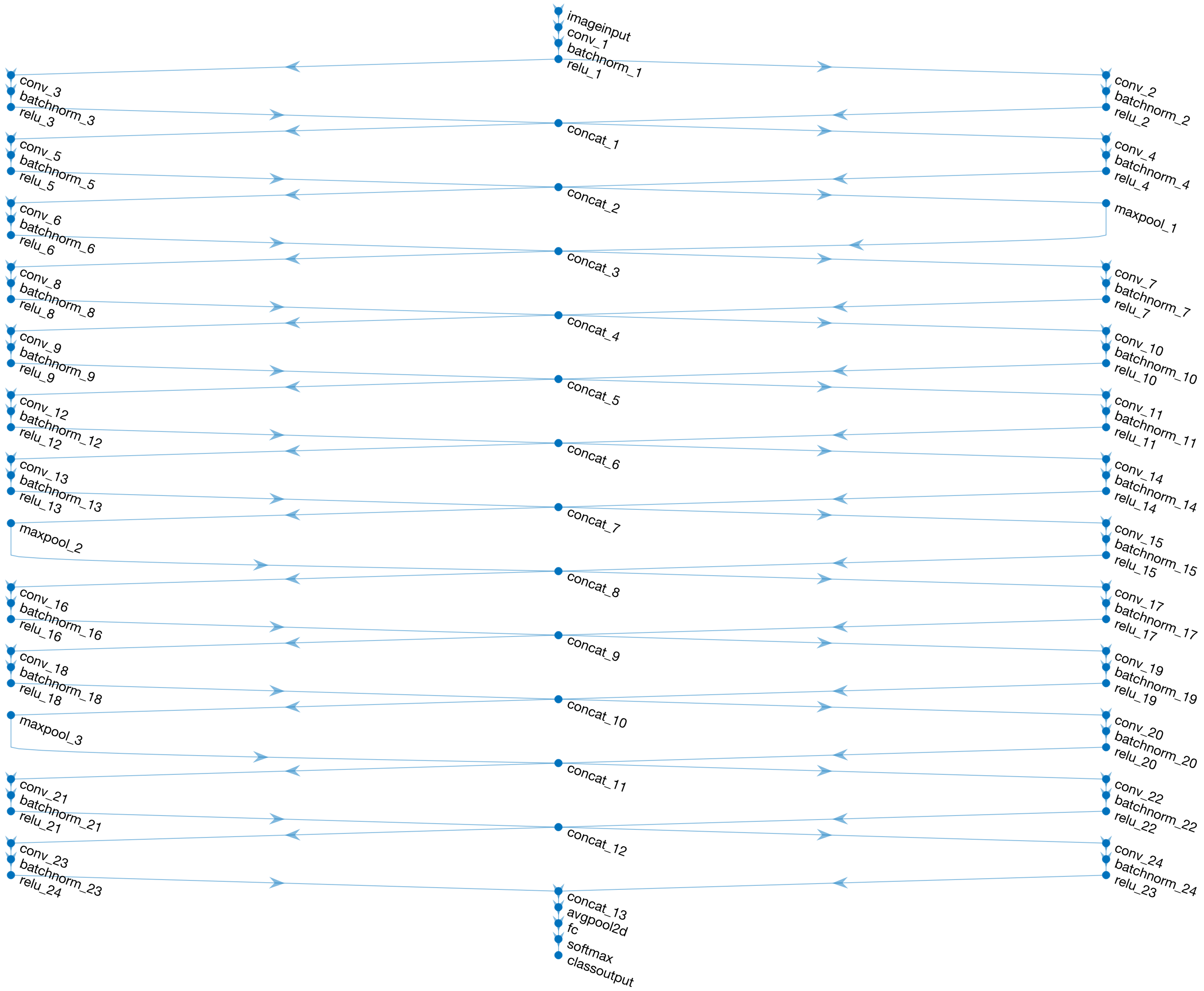

Supp. Figure 1 DeepFlow neural network architecture schematic. The DeepFlow network utilises a 64x64x2 input layer (x, y, channels) followed by repeating dual-path subunits from the "Inception" architecture to aggregate visual information over increasing scales. The number of kernels used increases at each layer, yielding 336 features maps with size 8 x 8 before average pooling, the fully connected (fc) layer and softmax classification using cross-entropy loss.

a

Balanced class weighting
Train = Cambridge & Cardiff, Test = GSK

Deep learning classification \ Human scorer classification	Binucleate	Binucleate +MN	Mononucleate	Mononucleate +MN	Other / unscorable	Tetranucleate	Tetranucleate +MN	Trinucleate	Trinucleate +MN	
Binucleate	**2244** 44.4%	**12** 0.2%	**23** 0.5%	**4** 0.1%	**20** 0.4%	**3** 0.1%	**0** 0.0%	**35** 0.7%	**0** 0.0%	95.9% 4.1%
Binucleate +MN	**28** 0.6%	**94** 1.9%	**1** 0.0%	**0** 0.0%	**6** 0.1%	**3** 0.1%	**0** 0.0%	**2** 0.0%	**3** 0.1%	68.6% 31.4%
Mononucleate	**161** 3.2%	**1** 0.0%	**1189** 23.5%	**7** 0.1%	**22** 0.4%	**0** 0.0%	**0** 0.0%	**0** 0.0%	**0** 0.0%	86.2% 13.8%
Mononucleate +MN	**4** 0.1%	**19** 0.4%	**17** 0.3%	**41** 0.8%	**83** 1.6%	**0** 0.0%	**0** 0.0%	**1** 0.0%	**0** 0.0%	24.8% 75.2%
Other / unscorable	**97** 1.9%	**7** 0.1%	**69** 1.4%	**7** 0.1%	**559** 11.0%	**5** 0.1%	**0** 0.0%	**17** 0.3%	**0** 0.0%	73.5% 26.5%
Tetranucleate	**1** 0.0%	**0** 0.0%	**0** 0.0%	**0** 0.0%	**2** 0.0%	**67** 1.3%	**0** 0.0%	**9** 0.2%	**0** 0.0%	84.8% 15.2%
Tetranucleate +MN	**0** 0.0%	**0** 0.0%	**0** 0.0%	**0** 0.0%	**8** 0.2%	**5** 0.1%	**1** 0.0%	**0** 0.0%	**0** 0.0%	7.1% 92.9%
Trinucleate	**54** 1.1%	**5** 0.1%	**0** 0.0%	**0** 0.0%	**9** 0.2%	**20** 0.4%	**0** 0.0%	**47** 0.9%	**0** 0.0%	34.8% 65.2%
Trinucleate +MN	**3** 0.1%	**4** 0.1%	**0** 0.0%	**0** 0.0%	**21** 0.4%	**2** 0.0%	**5** 0.1%	**8** 0.2%	**4** 0.1%	8.5% 91.5%
	86.6% 13.4%	66.2% 33.8%	91.5% 8.5%	69.5% 30.5%	76.6% 23.4%	63.8% 36.2%	16.7% 83.3%	39.5% 60.5%	57.1% 42.9%	**83.9% 16.1%**

b

Simplification to six classes
Train = Cardiff & Cambridge, Test = GSK

Deep learning classification \ Human scorer classification	Binucleates	Binucleates with MN	Mononucleates	Mononucleates with MN	Other or Unscorable	Polynucleates	
Binucleates	**2521** 49.8%	**2** 0.0%	**20** 0.4%	**6** 0.1%	**11** 0.2%	**14** 0.3%	97.9% 2.1%
Binucleates with MN	**3** 0.1%	**123** 2.4%	**0** 0.0%	**2** 0.0%	**1** 0.0%	**5** 0.1%	91.8% 8.2%
Mononucleates	**5** 0.1%	**0** 0.0%	**1139** 22.5%	**2** 0.0%	**6** 0.1%	**0** 0.0%	98.9% 1.1%
Mononucleates with MN	**0** 0.0%	**2** 0.0%	**6** 0.1%	**38** 0.8%	**0** 0.0%	**0** 0.0%	82.6% 17.4%
Other or Unscorable	**60** 1.2%	**10** 0.2%	**134** 2.6%	**10** 0.2%	**709** 14.0%	**12** 0.2%	75.8% 24.2%
Polynucleates	**3** 0.1%	**5** 0.1%	**0** 0.0%	**1** 0.0%	**3** 0.1%	**206** 4.1%	94.5% 5.5%
	97.3% 2.7%	86.6% 13.4%	87.7% 12.3%	64.4% 35.6%	97.1% 2.9%	86.9% 13.1%	**93.6% 6.4%**

Supp. Figure 2 Cross validation testing using class weighting or class simplification strategies. **a/b** Confusion matrices comparing human scoring versus deep learning image classifications for a test set of ~ 5000 unseen images. In each instance, the results reflect the outputs after training using image data from both the Cambridge and Cardiff laboratories before cross validation on new imaging cytometry data acquired at a third laboratory (GSK). In **a** class weighted cross entropy loss was used at the classification layer in an attempt to improve performance with the sparsely-represented phenotypes (*i.e.,* tri and tetranucleates with or without micronucleus (MN) events). In **b** these sparse, multinucleated categories were combined together into a single 'polynucleated' class. Whilst some improvements were realised using these strategies, they both reduced achieved accuracies (indicated, red squares) with one or more of the four, core phenotypes central to successful CBMN scoring (*i.e.,* mono or binucleated cells with or without MN events).

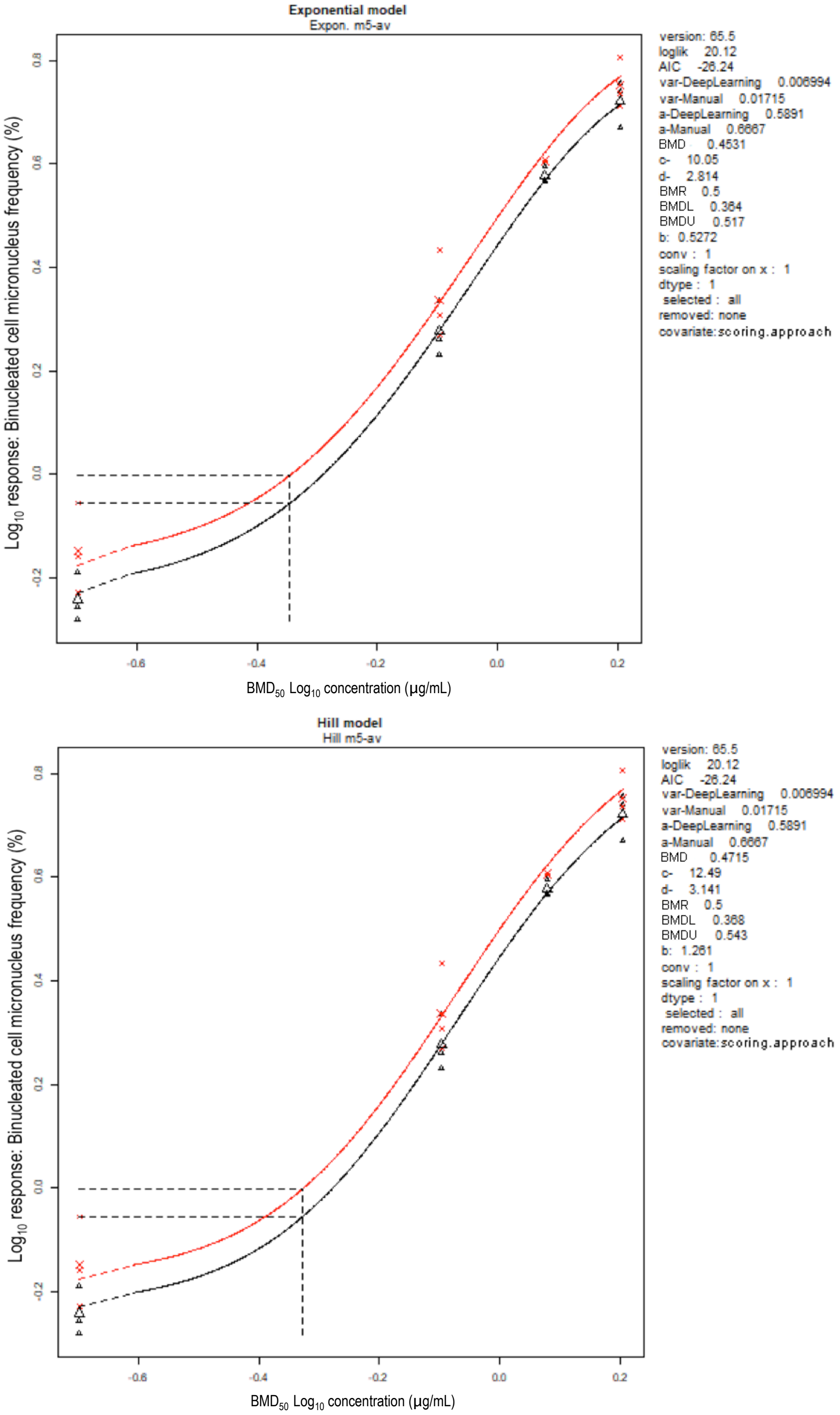

Supp. Figure 3 Benchmark dose (BMD) analysis using exponential and Hill model families. The curves represent fits to micronucleus concentration-response data obtained either by human (red) or neural network (black) scoring using either the exponential (top) or the Hill (bottom) model families. Both models were fitted with covariate (scoring method) dependent parameters for the background (parameter *a*) and within-group variance (*var*), whilst constant parameters could be used for potency, shape and steepness (parameters *b*, *c* and *d*). Horizontal and vertical dashed lines represent interpolation at a benchmark response (BMR) size of 50% to determine the BMD_{50} (respectively).
